# Supplementary material for: The Cortical Bone Metabolome of C57BL/6J Mice Is Sexually Dimorphic
Source: JBMR Plus. 2022 Jun 22;6(7):e10654. doi: 10.1002/jbm4.10654 (PMC9289981; doi:10.1002/jbm4.10654)
Supplement: Supplementary file 1 — Appendix S1 Table S1. Serum biomarkers, P1NP and CTX1, concentrations for different experiment groups. Table S2. Trabecular and cortical microarchitecture and cortical geometry from μCT. Fig. S1. H&E‐stained sections to assess the adequacy of marrow flushing. Fig. S2. Correlation analysis between ultimate stress and serum biomarker concentrations. [file JBM4-6-e10654-s001.docx]

## Supplementary Materials

**Note:** Source mass spectrometry files are available on the Metabolomics Workbench under Study ID ST002146 and ST002147.

**Supplementary Table 1.** Serum biomarkers, P1NP and CTX1, concentrations for different experiment groups. Data are presented as mean ± standard deviation from the mean. Ct. Th = cortical thickness (mm).

**Supplementary Table 2.** Trabecular and cortical microarchitecture and cortical geometry from microCT. Data are presented as mean ± standard deviation from the mean. BV/TV = bone volume/total volume; BMD = bone mineral density; Conn.D = connective density; SMI = structural model index; BS/BV = bone surface to bone volume; Tb.Th = trabecular thickness; Tb.N = trabecular number; Tb.S = trabecular spacing; Ct. TMD = cortex tissue mineral density; Ct. Area = cortical area; Imin = minimum moment of inertia; Ct. Th = cortical thickness; pMOI = polar moment of inertia; Tt. Area = Total Area; Ct. Area/ Tt. Area = Cortical Area/Total Area; Ma. Area = medullary area.

**Supplementary Table 3.** Raw metabolomics data. See attached.


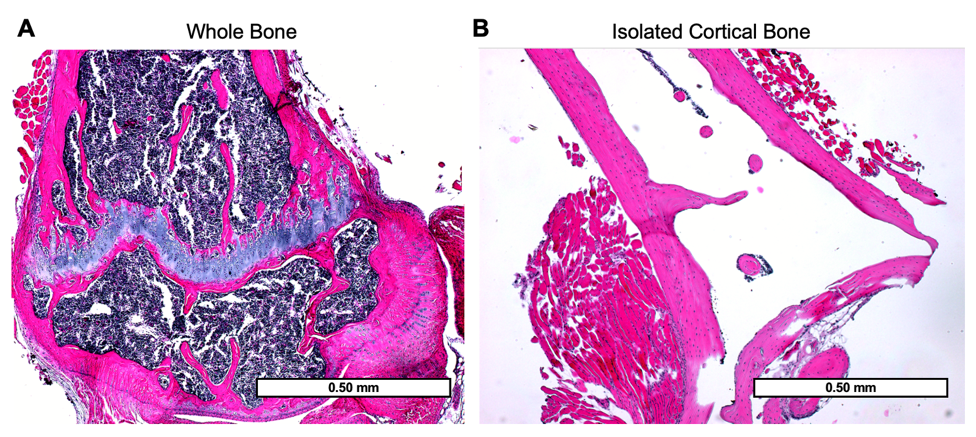


**Supplementary Figure 1. H&E stained sections to assess the adequacy of marrow flushing** (A) Whole bone and (B) marrow-flushed isolated cortical bone H&E-stained humeri imaged at 4x.


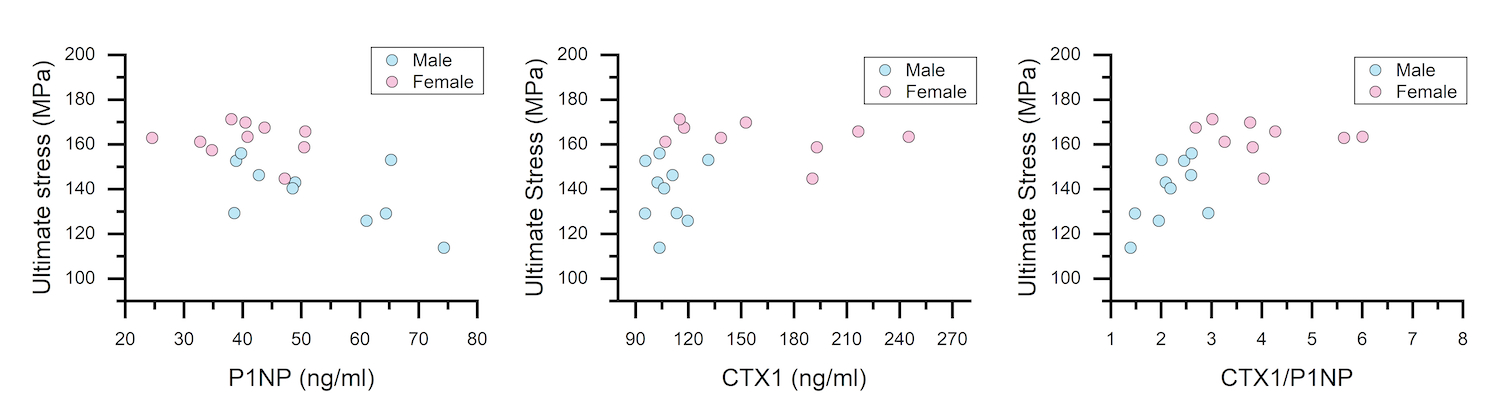


**Supplementary Figure 2. Correlation analysis between ultimate stress and serum biomarker concentrations.** (A) For pooled male and female data, there is a significant negative correlation between P1NP and ultimate stress, and a significant positive correlation between ultimate stress and (B) CTX1 and (C) CTX1/P1NP.

## Supplementary Methods

1. **Description of Statistical Analyses performed for untargeted metabolomic profiling.**

Untargeted metabolomic profiling is performed to analyze all metabolite features detected in samples. This often yields large datasets, therefore, univariate, supervised, and unsupervised multivariate analyses can be performed to best visualize and narrow the dataset. A commonly used platform to analyze metabolomic data is MetaboAnalyst. Here, raw data can be normalized, transformed, and scaled. Specifically, normalization is recommended to adjust for systematic differences among samples. Data transformation, such as log or square root transformation, can be executed. Finally, scaling allows for adjustment of features based on dispersion of the variable of interest.

Depending on the number of experimental groups and comparisons of interest, various analyses, unsupervised and supervised, can be performed. Commonly, hierarchical cluster analysis (HCA), principal component analysis (PCA), partial least squares-discriminant analysis (PLS-DA), variable importance in projection (VIP) score, dendrograms, volcano plot analysis, t-test, and fold change can be utilized to visualize and analyze data.

Unsupervised multivariate statistical analyses that are commonly utilized to visualize metabolomic data are HCA and PCA. HCA builds tree structures based on data similarities. With this information, one can visualize metabolomic profiles, identify potential subgroups within experimental groups, and overall, visualize potential differences that may exist between groups of interest. To further examine metabolic data in an unsupervised way, PCA can be employed. In this statistical analysis, data is linearly transformed, and the large dataset is reduced into latent variables – principal components (PCs). PCs are a combination of metabolite features that explain the variability in the dataset. For example, a PC percentage of 18.2% suggests that 18.2% of the variability in the dataset is accounted for. Therefore, PCs allow researchers to gauge variability and distinguish the presence or absence of separation between groups.

A common next step following PCA is to perform PLS-DA, a supervised analysis. PLS-DA is like PCA as it helps visualize differences between cohorts but to do so, it uses a linear regression model and projects the predicted and observed variables. An extension of PLS-DA is VIP scores, which are useful to metabolomics because metabolites are scored based on their contribution to discrimination between groups. By performing these few statical methods, HCA, PCA, PLS-DA, and VIP scores initially provide an encompassing view of the similarities and differences between comparison groups of interest.

To further visualize data and begin to identify metabolite features, or groups of metabolite features, that are unique to one group, analyses like fold change and volcano plots can be performed. Implementing both tests when analyzing metabolomics data allows for metabolites that differ in intensity between groups to be elucidated. Volcano plot is a common test applied to analyze metabolomic data because it shows statistical significance vs. magnitude of change. This visual displaying p-value and fold change together enables metabolite features that have large fold changes, are statistically significant, and belong to one experimental group but not the other to be identified. By identifying features that meet this criteria, biological relevance can be investigated. Specifically, differentially expressed metabolites can be subjected to pathway analysis using MetaboAnalyst’s MS Peaks to Pathways feature using the *mummichog* algorithm. This tool is able to take the metabolite compounds identified via the statistical tests describes to be associated with biological pathways. With this information, networks of functional cellular activity and differences in activity between groups can be investigated further.
